# Outcomes of prostate cancer screening among men using antidiabetic medication

**DOI:** 10.1038/s41598-021-86534-2

**Published:** 2021-04-01

**Authors:** A. Vettenranta, T. J. Murtola, K. Talala, K. Taari, U.-H. Stenman, T. L. J. Tammela, A. Auvinen

**Affiliations:** 1 Faculty of Medicine and Life Sciences, University of Tampere, Arvo Ylpön Katu 34, PO Box 100, 33014 Tampere, Finland; 2 Department of Urology, Tampere University Hospital, Tampere, Finland; 3 Finnish Cancer Registry, Helsinki, Finland; 4 Department of Urology, Helsinki University Hospital, University of Helsinki, Helsinki, Finland; 5 Department of Clinical Chemistry, University of Helsinki, Helsinki, Finland; 6 Faculty of Social Sciences, University of Tampere, Tampere, Finland

**Keywords:** Predictive markers, Epidemiology, Prostate

## Abstract

Diabetic men have decreased risk for prostate cancer (PCa) overall and lower PSA compared to non-diabetics. This may affect the outcomes of PSA-based screening. We investigated the effect of PSA-based screening at 4-year intervals on PCa incidence and mortality separately among users and non-users of antidiabetic medication with the hypothesis that screening would detect less low-grade cancer and more high-grade cancer in diabetic men. A cohort of 80,458 men from the Finnish Randomized Study of Screening for Prostate Cancer (FinRSPC) were linked to national prescription database to obtain information on antidiabetic medication purchases. PCa risk and mortality were compared between the FinRSPC screening arm (SA) and the control arm (CA) separately among users and non-users of antidiabetic medication. Among antidiabetic medication users median PSA was lower than in non-users (0.93 and 1.09 ng/ml, respectively, P for difference = 0.001). Screening increased overall PCa incidence compared to CA after the first screen both among medication users and non-users (HR 1.31, 95% CI 1.08–1.60 and HR 1.55, 95% CI 1.44–1.66, respectively). On the second and third screen the difference between SA and CA attenuated only among medication users. Detection of Gleason 6 tumors was lower among medication users, whereas no difference was observed in detection of Gleason 8–10 cancers. Concordantly, screening affected PCa mortality similarly regardless of antidiabetic medication use (HR 0.38, 95% CI 0.14–1.07 and HR 0.19, 95% CI 0.11–0.33 among users and non-users after three screens, respectively. *P* for difference = 0.18). Median PSA is lower in men using antidiabetic drugs than among non-users. Systematic PSA screening detects less low-risk tumors among medication users, whereas detection of high-risk tumors and mortality effects are similar regardless of medication use. This suggests that antidiabetic medication users may form a suitable target group for PCa screening, with less screening-related overdiagnosis of indolent tumors.

## Introduction

Prostate-specific antigen (PSA) based screening for prostate cancer (PCa) remains controversial. Screening may reduce PCa mortality but also causes unnecessary over-diagnosis of clinically insignificant tumours and ensuing overtreatment^1^. The Finnish Randomized Study of Screening for Prostate Cancer (FinRSPC), the largest component of the European Randomized Study of Screening for Prostate Cancer (ERSPC) showed a small, statistically non-significant reduction in prostate cancer-specific mortality by systematic PSA-based screening^1,2^.

Men with diabetes mellitus (DM) have lower overall PCa incidence compared to non-diabetic men, though the reason behind the association is unclear^3^. The risk decrease may be driven by lowered risk of low-grade prostate tumors, whereas the risk of high-grade and advanced tumors is increased. One likely reason for such discrepancy by tumor type is lowered PSA among diabetic men; men using antidiabetic medication have lower blood PSA level than healthy controls^3,4^. Diabetic men not requiring medication have similar PSA values to non-diabetic men^4^. Lower PSA among diabetic men would mean less biopsies for elevated PSA, which could explain lowered incidence of low-grade tumors. High-grade tumors, on the other hand, are often symptomatic and thus detection is less dependent on PSA.

Metformin use, the most common drug for type two diabetes, has been linked with lowered PSA in men with castration-resistant PCa^5^, but not all studies agree^6,7^. Like diabetes, metformin use has been associated with decreased PCa risk^8^. Diabetic men receiving insulin treatment have been found to have 39% lower blood PSA levels compared to non-diabetic men^4^. However, it is not known how this affects the outcomes of PSA-based prostate cancer screening.

We compared the effect of PSA-based screening on PCa risk between FinRSPC screening arm and control arm among users and nonusers of antidiabetic medication. Effect of screening on risk of PCa overall and stratified by Gleason grade and tumor extent, as well as risk of PCa death was explored by use of antidiabetic medication.

## Materials and methods

### The study cohort

The study population included 80,458 men from FinRSPC, the largest component of the multicenter ERSPC^1,2^. In 1996–1999, all men aged 55, 59, 63 or 67 and living at the Tampere or Helsinki area were identified from the Population Registry of Finland. Men with prevalent prostate cancer diagnosis, PCa deaths and those who emigrated were excluded at baseline. Remaining men (80,139) were randomized into two trial arms: screening arm (SA) of 31,866 men and control arm (CA) of 48,273 men.

Screening was performed in three subsequent rounds. First round in 1996–1999, second in 2000–2003 and third in 2004–2007. Men in the SA were invited to be screened at four year intervals. Invitations stopped at PCa diagnosis, emigration from the study area or upon age of 71. Men aged 67 at the start of the study were invited for screening twice.

Men with PSA greater or equal of 4.0 ng/l were considered screen-positive and referred to their local urological clinic for diagnostic examinations which included digital rectal examination (DRE), transrectal ultrasound and prostate biopsy. Men with PSA of 3.0–3.9 ng/l were referred to additional testing which in 1996–1998 was DRE and since 1999 free PSA ratio with 16% cut-off point.

Information on PCa cases in both trial arms was obtained from the comprehensive nation-wide Finnish Cancer Registry. Clinical information was collected from medical records. Data includes information about PCa cases, date of diagnosis, Gleason grade, TNM-stage and primary treatment.

In Finland all deaths are registered by cause of death committee maintained by Statistics Finland. The information is collected from mandatory death certificates. The FinRSPC cause of death committee validated the accuracy of recorded PCa deaths by comparing the recorded diagnoses with medical records. Process was blinded in terms of official cause of death and trial arm. Deaths and PCa cases are included until the end of year 2015.

The study complied with Declaration of Helsinki. Informed consent was obtained from men in the screening arm of the study, Men in the control arm were followed via national registries and were not contacted personally, thus informed consent was not necessary.

### Information about antidiabetic medication

Information on usage of antidiabetic medication from 1996 until 2009 was obtained from the prescription database of the Social Insurance Institution (SII) of Finland. Medication use was obtained for this time period as FinRSPC screening intervention took place at this time. Data included details about the date of purchase, dose, amount of doses and amount of packages purchased. Information about medications use was available for 97.7% of the FinRSPC study population (78,615 men). SII is a governmental agency operating under the Ministry of Health. Finnish residents receive reimbursement for outpatient purchases for physician-prescribed medication as part of national health insurance which covers all citizens of Finland^9^. Most commonly the reimbursement is received as a price discount at the pharmacy. All reimbursed purchases are recorded by the SII prescription database. Over-the-counter purchases and drug usage at hospital inpatient periods are not recorded. In Finland antidiabetic drugs are available by physician prescription only, thus comprehensively recorded by the registry.

Only medication purchases occurring before PCa diagnosis, emigration or end of 2009 was included in the analysis. Each year with recorded purchases of any antidiabetic drugs, regardless of the amount, was counted as year of use. Yearly amount of medication use was calculated by dividing the yearly total purchased mg/IU amount of antidiabetic medication with amount corresponding to a Defined Daily Dose (DDD) of the drug^10^ and adding together DDDs of separate antidiabetic drugs. Intensity of medication use was evaluated by dividing the cumulative number of DDDs with cumulative years of usage before the analysed screening round. Analysis was stratified by median of the intensity of usage (DDDs/year) to estimate dose-dependence.

### Statistical analysis

Cox proportional hazards regression was used to analyse hazard ratios (HRs) and 95% confidence intervals (95% CIs) for PCa risk and mortality by trial arm. (Supplementary Table 3. Flowchart).

The exposure of interest was PSA-based PCa screening. The FinRSPC screening arm was compared to control arm for PCa risk and PCa death. Analyses were stratified by use of antidiabetic medication.

Median PSA and Free/Total PSA percentage were analysed by each screening round separately for overall DM medication use and for metformin users and non-users. Only medication usage that occurred before each screening round was included in the analyses on PSA. The values in the second and third round were subtracted from the value from the previous screening round to obtain PSA change. Median PSA change was compared by antidiabetic drug use. Statistical significance of differences in median PSA and Free/total PSA percentage on each screening round and changes between the rounds was analysed with Mann–Whitney U test.

PCa risk and mortality were analyzed by the number of screenings attended to assess whether the role of antidiabetic medication as modifier of effects of PSA-based screening changes by subsequent screening rounds. Men randomized to the screening arm were categorized according to the number of screening rounds attended on a given follow-up time point (one, two, three or none). 5726 men attended screening once, 7716 twice and 10,328 three times. From men attending screening once, twice or three times, users of antidiabetic medication were 1071, 1547 and 2052, respectively. The screening round variable was updated separately for each follow-up month according to the date of screening tests. A time-dependent covariate was created and included in the Cox regression model. Men in the control arm were used as the reference. Men randomized in 1996 were excluded from the analysis 31.12.2012, men randomized 1997 were excluded 31.12.2013, men randomized 1998 were excluded 31.12.2014 and men randomized 1999 were excluded 31.12.2015. The maximum follow up time was 17 years. PCa diagnosis, PCa death or emigration from the study area stopped the follow up time. The risk of PCa was analyzed overall, by Gleason score (categorized as Gleason 6, 7, 8–10) and by tumor TNM stage (categorized as localized disease; T1-T3aNx/0Mx/0 or advanced T3b-T4, all N1, all M1 cases).

All analyses were performed for antidiabetic drugs overall, by intensity of usage and separately for metformin. Effect modification by overall antidiabetic medication usage was evaluated by adding an interaction term between antidiabetic medication usage and the trial arm to Cox regression model.

Effect of screening on PCa mortality between antidiabetic medication users and non-users was analyzed with Cox regression model, similarly as the analyses for PCa risk. Only medication use prior PCa diagnosis was included, since we investigated screening outcomes, not direct risk by use of antidiabetic medication.

All statistical tests are two-sided. Analyses were carried out using IBM SPSS 24 (Chicago Illinois, US) and Stata statistical software (College Station Texas, US).

### Ethical approval

The study was approved by ethical committee of the Pirkanmaa Hospital District (Decision R10167).

## Results

### Baseline characteristics

Information on DM medication use was available for 78,615 men (SA 31,866 men CA 48,273 men). Of these 6,144 were antidiabetic medication users in SA and 9,749 in CA. Median age at randomization was 59 years in both trial arms, with no difference by antidiabetic medication use. Percentage of PCa cases was slightly lower among antidiabetic medication users in both trial arms. Median BMI and Charlson comorbidity score were higher among antidiabetic medication users compared to non-users (Table 1).

**Table 1:** Descriptive characteristics. Study population of 78,615 men from the Finnish Randomized Study of Prostate Cancer Screening.

	FinRSPC study arm			
	Screening arm		Control arm	
	Antidiabetic drug users	Antidiabetic drug non-users	Antidiabetic drug users	Antidiabetic drug non-users
Number of participants	6144	24,050	9749	38,672
Age at randomization; median (IQR)	59 (55–63)	59 (55–63)	59 (55–63)	59 (55–63)
Years of follow-up until PCa diagnosis; median (IQR)	17 (11.50–16.80)	17 (10.40–17.00)	17 (11.50–16.30)	17 (11.20–17.00)
Years of follow-up until death; median (IQR)	17 (12.80–17.00)	18 (14.00–17.00)	17 (12.70–17.00)	17 (14.00–17.00)
N of prostate cancer diagnoses	727 (11.8%)	3067 (12.8%)	1030 (10.6%)	4437 (11.5%)
**Tumor grade at diagnosis**				
Gleason 6 or less; n (% of diagnoses)	392 (53.9%)	1699 (55.4%)	407 (39.5%)	1854 (41.8%)
Gleason 7; n(%)	176 (24.2%)	772 (25.2%)	343 (33.3%)	1401 (31.6%)
Gleason 8–10; n (%)	120 (16.5%)	404 (13.2%)	215 (20.9%)	802 (18.1%)
Unknown	36 (5.4%)	192 (6.3%)	65 (6.3%)	380 (8.6%)
Tumor T-stage at diagnoses				
T1/T2	576 (79.2%)	2,468 (80.5%)	725 (70.4%)	3194 (72%)
T3	104 (14.3%)	349 (11.4%)	208 (20.2%)	770 (17.4%)
T4	17 (2.3%)	78 (2.5%)	49 (4.8%)	202 (4.6%)
Unknown	30 (4.1%)	172 (5.6%)	48 (4.7%)	271 (6.1%)
Tumor M-stage at diagnosis:				
Localized (M0/x); n (%):	655 (90.1%)	2764 (90.1%)	898 (87.2%)	3,832 (66.4%)
Metastatic (M1); n (%)	45 (6.2%)	146 (4.8%)	92 (8.9%)	359 (8.1%)
Unknown	27 (3.7%)	157 (5.1%)	40 (3.9%)	246 (5.5%)
*n* (%) of deaths				
Overall	2,703 (44%)	8,323 (34.6%)	4,273 (43.8%)	13,637 (35.3%)
Prostate cancer-specific	78 (1.3%)	245 (1.0%)	112 (1.1%)	489 (1.3%)
Body Mass Index (kg/m^2^), median (IQR)	28 (26–31)	26 (24–28)	–	–
Median Charlson comorbidity score (IQR)	2 (1–3)	0 (0–1)	2 (1–3)	0 (0–1)

### Effect of antidiabetic medication use on PSA

In all three screening rounds, median PSA and the percentage of screen-positive men were slightly lower among antidiabetic medication users versus nonusers. The same was observed for users of metformin. Median PSA change between the screening rounds was lower among the medication users (Table 2).

**Table 2:** Median PSA and change in PSA value between screening round among antidiabetic medication users and non-users. Study population of 78,615 men from the Finnish Randomized Study of Prostate Cancer Screening.

	1st screening round		2nd screening round			3rd screening round		
Initiation of antidiabetic medication use between screening rounds	Median PSA (95% IQR)	n (%) of screen-positive men	Median PSA (95% IQR)	Median PSA change from previous screening round (95% IQR)	n (%) of screen-positive men	Median PSA (95% IQR)	Median PSA change from previous screening round (95% IQR)	n (%) of screen-positive men
**All antidiabetic medication**								
No	1.09 (0.64–1.98)	1918 (10%)	1.33 (0.77–2.45)	0.26 (0.30–0.75)	2074 (12.5%)	1.46 (0.83–2.61)	0.19 (−0.06–0.69)	1422 (13.8%)
Yes	0.93 (0.55–1.70)	122 (7.8%)	1.18 (0.67–2.23)	0.21 (0.1–0.63)	194 (10.1%)	1.22 (0.69–2.26)	0.12 (−0.09–0.50)	180 (9.9%)
*P* value	0.001	0.005	0.000	0.001	0.003	0.000	0.000	0.000
**Metformin**								
No	1.08 (0.64–1.97)	1993 (9.9%)	1.32 (0.77–2.44)	0.26 (0.03–0.74)	2,159 (12.4%)	1.45 (0.82–2.60)	0.19 (−0.06–0.68)	1473 (13.0%)
Yes	0.89 (0.53–1.57)	47 (6.7%)	1.15 (0.65–2.18)	0.20 (0.10–0.62)	114 (10.0%)	1.19 (0.67–2.20)	0.11 (-0.10–0.48)	129 (9.4%)
*P* value	0.000	0.004	0.000	0.002	0.018	0.000	0.000	0.000

	Median free/total PSA %		Median free/total PSA %	Median change from previous screening round (95% IQR)		Median free/total PSA %	Median change from previous screening round (95% IQR)	
**All antidiabetic medication**								
None	26.3 (20–34.20)		26.10 (19.90–33.40)	−0.80 (−4.90–3.20)		28.40 (21.90–36.20)	1.90 (−2.50–6.60)	
Any	26.9 (20.5–36.00)		28.20 (21.20–36.10)	0.30 (−4.00–4.70)		31.60 (24.40–41.40)	3.70 (−1.00–8.80)	
*P* value	0.001			0.001		0.000		
**Metformin**								
No	26.30 (20.00–34.20)		26.20 (19.90–33.50)	−0.80 (−4.90–3.20)		28.50 (21.90–36.30)	2.00 (−2.50–6.70)	
Yes	26.80 (20.70–35.90)		28.20 (21.40–36.30)	0.80 (−3.70–5.45)		32.00 (24.80–41.90)	4.00 (−0.70–9.10)	
*P* value	0.026		0.000	0.000		0.000	0.000	

Median of percentage of the free/total PSA ratio was slightly higher among users of antidiabetic medication versus non-users in the first screening round. The difference came more pronounced in the following rounds. Median percentage of free/total PSA ratio increased more in antidiabetic medication users in subsequent rounds compared to non-users (Table 2).

### Effect of screening on PCa incidence among users and non-users of antidiabetic medication

#### Detection of low-grade/localized prostate cancer

Among men using antidiabetic medication the incidence of PCa overall was elevated after the first screening compared to the control arm (HR 1.31 95% CI 1.08–1.60 *P* for interaction < 0.001). The effect diminished in the subsequent screening rounds. Among medication non-users the incidence of PCa overall remained elevated in the SA compared to the CA in all screening rounds. (Table 3).

**Table 3:** Effect of PSA-based screening on incidence of prostate cancer overall and clinically non-significant disease as defined by Gleason score and TNM stage, stratified by antidiabetic medication use. Study population of 78,615 men from the Finnish Randomized Study of Screening for Prostate Cancer.

	PCa incidence, overall			Gleason 6 or less			Localized PCa		
	nro of screening rounds participated			nro of screening rounds participated			nro of screening rounds participated		
	1	2	3	1	2	3	1	2	3
	HR (95% CI)	HR (95% CI)	HR (95% CI)	HR (95% CI)	HR (95% CI)	HR (95% CI)	HR (95% CI)	HR (95% CI)	HR (95% CI)
**All antidiabetic medication**									
n of users/non-users	15,408/63,195	15,408/63,195	15,408/63,195	14,630/59,192	14,630/59,192	14,630/59,192	15,256/62,419	15,256/62,419	15,256/62,419
No antidm usage	1.55 (1.44–1.66)	1.22 (1.13–1.31)	1.31 (1.20–1.43)	2.00 (1.83–2.18)	1.82 (1.65–1.99)	1.93 (1.70–2.19)	1.61 (1.49–1.73)	1.30 (1.21–1.40)	1.42 (1.30–1.55)
Any antidm usage	1.31 (1.08–1.60)	0.89 (0.74–1.08)	0.98 (0.80–1.21)	1.67 (1.26–2.21)	1.35 (1.03–1.77)	1.74 (1.27–2.40)	1.31 (1.06–1.62)	0.97 (0.79–1.17)	1.06 (0.85–1.32)
*P* for interaction	< 0.001	0.013	0.83	0.011	0.19	0.21	< 0.001	0.026	0.88
**Intensity of medication use**									
Median or below	1.47 (1.12–1.92)	0.91 (0.70–1.19)	1.02 (0.75–1.38)	1.93 (1.32–2.81)	1.47 (1.01–2.12)	2.12 (1.37–3.26)	1.48 (1.11–1.97)	1.02 (0.78 -1.34)	1.08 (0.79–1.48)
Above median	1.17 (0.88–1.56)	0.87 (0.67–1.14)	0.95 (0.70–1.27)	1.43 (0.94–2.17)	1.23 (0.83–1.84)	1.40 (0.87–2.26)	1.15 (0.85–1.57)	0.91 (0.69–1.21)	1.03 (0.76–1.40)
*P* for interaction	0.16	0.87	0.96	0.19	0.48	0.34	0.15	0.66	0.98

When stratified by Gleason score screening increased detection of Gleason 6 tumors especially among non-users of anti-DM medication and less among anti-DM drug users. The findings were similar also for localized tumors.

Analyses in the subgroup of metformin users showed similar results as antidiabetic drugs overall (Supplementary Table 1).

#### Detection of high-grade/advanced prostate cancer

After 2 or 3 screenings men in SA generally had lowered risk of Gleason 7, Gleason 8–10 and advanced tumors compared to CA (Table 4). Among anti-DM medication users more Gleason 8–10 and advanced tumors were detected on the 1st screening, although the difference compared to non-users was non-significant.

**Table 4:** Effect of PSA-based screening on incidence on clinically significant prostate cancer as defined by Gleason score and TNM stage, stratified by antidiabetic medication use. Study population of 78,615 men from the Finnish Randomized Study of Screening for Prostate Cancer.

	Gleason 7			Gleason 8–10			Advanced PCa		
	nro of screening rounds participated			nro of screening rounds participated			nro of screening rounds participated		
	1	2	3	1	2	3	1	2	3
	HR (95% CI)	HR (95% CI)	HR (95% CI)	HR (95% CI)	HR (95% CI)	HR (95% CI)	HR (95% CI)	HR (95% CI)	HR (95% CI)
**All antidiabetic medication**									
n of users/non-users	14,576/57,654	14,576/57,654	14,576/57,654	14,570/56,570	14,570/56,570	14,570/56,570	14,284/55,980	14,284/55,980	14,284/55,980
No antidm usage	1.14 (0.99–1.33)	0.80 (0.70–0.92)	1.09 (0.94–1.26)	1.02 (0.83–1.27)	0.70 (0.57–0.85)	0.83 (0.67–1.03)	1.04 (0.81–1.34)	0.49 (0.36–0.67)	0.50 (0.34–0.73)
Any antidm usage	0.90 (0.61–1.34)	0.66 (0.47–0.94)	0.70 (0.48–1.01)	1.38 (0.92–2.06)	0.56 (0.36–0.88)	0.66 (0.41–1.04)	1.36 (0.80–2.33)	0.33 (0.14–0.76)	0.44 (0.19–1.01)
*P* for interaction	0.10	0.29	0.17	0.52	0.42	0.72	0.65	0.37	0.86
**Intensity of medication use**									
Median or below	1.09 (0.64–1.86)	0.65 (0.40–1.07)	0.67 (0.38–1.18)	1.32 (0.73–2.39)	0.47 (0.24–0.95)	0.47 (0.23–0.98)	1.41 (0.68–2.94)	0.20 (0.05–0.81)	0.59 (0.21–1.67)
Above median	0.73 (0.40–1.34)	0.68 (0.42–1.10)	0.71 (0.43–1.18)	1.42 (0.81–2.48)	0.65 (0.36–1.17)	0.86 (0.47–1.56)	1.33 (0.61–2.90)	0.50 (0.18–1.41)	0.29 (0.07–1.22)
*P* for interaction	0.26	0.98	0.65	0.87	0.34	0.30	0.90	0.33	0.52

### Effect of screening on PCa mortality by use of antidiabetic medication

Antidiabetic medication users screened once had higher PCa mortality compared to the CA (HR 2.56; 95% CI 1.50–4.38). After the first screen PCa mortality was elevated also among non-users, though slightly less (HR 1.53; 95% CI 1.22–1.93) (Table 5). After second and third screening PCa mortality was lower among men in the screening arm both among antidiabetic medication users and non-users, with no statistically significant difference by medication use.

**Table 5:** Effect of screening on prostate cancer—specific mortality among users and non-users of antidiabetic medication. Study population of 78,615 men from the Finnish Randomized Study of Screening for Prostate Cancer.

Any antidiabetic medication use	Mortality by antidiabetic medication usage HR (95% CI)		
Number of participated screening rounds	1	2	3
No antidm usage	1.53 (1.22–1.93)	0.52 (0.39–0.70)	0.19 (0.11–0.33)
Any antidm usage	2.56 (1.50–4.38)	0.42 (0.18–0.98)	0.38 (0.14–1.07)
P for interaction	0.11	0.60	0.18
**Intensity of medication use**			
Median or below	2.08 (0.95–4.54)	0.24 (0.06–1.01)	na
Above median	3.20 (1.52–6.72)	0.67 (0.23–1.93)	0.84 (0.29–2.44)
*P* for interaction	0.46	0.28	na

### Effects of screening by intensity of medication used

Prostate cancer risk or mortality in the screening arm compared to CA was not significantly modified by intensity of antidiabetic medication usage (Tables 3, 4, 5).

## Discussion

In our large population-based randomized screening trial median PSA was lower among antidiabetic medication users compared to the non-users in all three screening rounds. In the first screening round this difference was 15% and continued to increase for subsequent rounds. Thus, smaller proportion of diabetic men were screen positive.

As a consequence, diabetes modified effects of PSA-based PCa screening. The results suggest a modest decline in detection of clinically insignificant cancers in the SA among men using antidiabetic medication compared to the screened men of anti-DM medication non-users. Furthermore, men using anti-DM medication seem to have increased detection of Gleason 8–10 and advanced PCa especially when screened for the first time compared to non-users. Diabetic men also had significantly higher risk for PCa death when screened for the first time. This might be due the higher prevalence of high-grade and advanced cancers among diabetic men^11,12^ that are in high-risk of progression to fatal disease. The risk of PCa death decreased in the screening arm compared to the control arm in the subsequent screenings regardless of anti-DM drug use.

Besides lowered PSA, another potential explanation for lower incidence of low-grade cancer among anti-DM drug users may be opportunistic PSA-screening being more common. Men with long-term conditions such as diabetes, participate more often in PSA-based screening for prostate cancer as part of regular health-care contact than men without such conditions^13,14^. However, strongest effect of screening on elevated PCa incidence was observed in the first screening round both in users and non-users of antidiabetic medication. This does not support extensive opportunistic screening among anti-DM drug users, as systematic screening program still has effect on PCa incidence also in this group.

Previously, metformin use has been associated with lowered PSA in some studies^15,16^. In our study, median PSA values were lowered compared to non-users similarly among users of metformin as among anti-DM drug users in general.

Our findings of lower PSA among antidiabetic medication users compared to non-users align with previous studies^3,4,17–19^. Many possible explanations behind this phenomenon have been investigated. Obesity has similarly been associated with lowered PSA^20^. Overweight and diabetic men have higher body mass and thus higher plasma volume. Higher plasma volume may dilute PSA^21^. Diminished PSA in overweight men may also be confounded by numerous other factors such as medication use, age, and prostatic hyperplasia^22^. Age- and BMI-adjusted PSA levels have not been found to be more useful than non-adjusted PSA in PCa screening^23^, which supports the multifactorial nature of PSA levels.

Previous studies have reported decreased overall PCa risk among diabetic men compared to non-diabetic men^3,24,25^. Lower PSA levels in diabetic men may lead to less prostate biopsies due to elevated PSA, reducing the incidence of PSA-detected PCa, as noted in this study. Similarly, diabetic men have been reported to harbor more often high-grade PCa compared to non-diabetic men at similar PSA level^11,12^. Therefore previous seemingly paradoxical results on lower overall PCa risk but increased risk of high-grade cancer among diabetic men may be due to less detection of indolent low-grade disease based on PSA elevations, which leads to higher proportion of diagnosed tumor to be high-grade disease often detected by symptoms.

However, association between PCa risk and diabetes appears to depend on definition of diabetes; in FinRSPC antidiabetic medication is associated with lowered overall PCa risk but increased risk of metastatic disease^26^, whereas men with hyperglycemia, i.e. diabetes blood glucose level have increased risk of PCa compared to normoglycemic men regardless of tumor grade^27^. Thus, diabetes itself, especially hyperglycemia, appears to be a PCa risk factor, whereas antidiabetic medication use mitigates the risk increase^27^.

Strengths of our study come from the opportunity to evaluate the effects of systematic randomized PSA screening effort in a population-based cohort of men followed for a long period of time. This gives our study statistical power and removes selection bias and influence of confounding factors by ensuring equal distribution of background variables. Information about medication use is exceptionally detailed and accurate, as is information on PCa cases and deaths.

A weakness of our study is that we only used information about antidiabetic medication usage as on marker of diabetes. This does not include undiagnosed diabetes or men treated with diet and lifestyle intervention only, causing bias towards the null. Opportunistic screening became more popular in the Finnish population during FinRSPC trial^28^. This is likely to be more common in diabetic men due to more frequent health-care contacts^12^, diminishing the effect of systematic screening effort on PCa incidence and death within this group.

In summary, we found mean PSA values to be lower and PSA progression slower among users of antidiabetic medication compared to non-users. Compared to control arm with no systematic screening, systematic PSA-based screening increases detection of localized prostate tumors less among diabetic men than among non-diabetic men. Nevertheless, detection of high-grade or advanced tumors and effects of screening on prostate cancer mortality are similar regardless of antidiabetic medication use. Diabetic men may be a suitable target group for PSA-based screening, as screening causes less overdiagnosis of low-risk disease among them while the mortality benefits from screening do not differ compared to non-diabetic men.

## Supplementary Information

Supplementary Information 1.

Supplementary Information 2.

Supplementary Information 3.
