# Supplementary material for: Outcomes of prostate cancer screening among men using antidiabetic medication
Source: Sci Rep. 2021 Apr 1;11:7363. doi: 10.1038/s41598-021-86534-2 (PMC8016840; doi:10.1038/s41598-021-86534-2)
Supplement: Supplementary file 1 — Supplementary Information 1. [file 41598_2021_86534_MOESM1_ESM.docx]

Outcomes of prostate cancer screening among men using antidiabetic medication

Vettenranta A^1^, Murtola TJ^1,2^, Talala K^3^, Taari K^4^, Stenman U-H^4,5^, Tammela TLJ^1,2^, Auvinen A^6^

^1^ University of Tampere, Faculty of Medicine and Life Sciences, Tampere, Finland

^2^ Tampere University Hospital, Department of Urology, Tampere, Finland

^3^ Finnish Cancer Registry, Helsinki, Finland

^4^ Department of Urology, University of Helsinki and Helsinki University Hospital, Helsinki, Finland

^5^ Department of Clinical Chemistry, University of Helsinki, Helsinki, Finland

^6^ University of Tampere, Faculty of Social Sciences, Tampere, Finland

Corresponding author: Ms. Arla Vettenranta. Arvo Ylpön katu 34, PO box 100, 33014 University of Tampere, Finland. Phone: +358 3 355 111. E-mail: arla.vettenranta@tuni.fi

Table 3. Effect of PSA-based screening on incidence of prostate cancer overall and clinically non-significant disease as defined by Gleason score and TNM stage. Study population of 78,615 men from the Finnish Randomized Study of Screening for Prostate Cancer

|  | PCa incidence, overall | | | Gleason 6 or less | | | Localized PCa | | |
| --- | --- | --- | --- | --- | --- | --- | --- | --- | --- |
|  | nro of screening rounds participated | | | nro of screening rounds participated | | | nro of screening rounds participated | | |
|  | 1 | 2 | 3 | 1 | 2 | 3 | 1 | 2 | 3 |
|  | HR (95% CI) | HR (95% CI) | HR (95% CI) | HR (95% CI) | HR (95% CI) | HR (95% CI) | HR (95% CI) | HR (95% CI) | HR (95% CI) |
| Metformin | |  |  |  |  |  |  |  |  |
| n of users/non-users | 12,258/66,345 | 12,258/66,345 | 12,258/66,345 | 11,672/62,150 | 11,672/62,150 | 11,672/62,150 | 12,143/65,532 | 12,143/65,532 | 12,143/65,532 |
| None | 1.54 (1.44 – 1.65) | 1.21 (1.13 – 1.30) | 1.33 (1.22 – 1.45) | 1.99 (1.83 – 2.18) | 1.81 (1.65 – 1.98) | 1.99 (1.76 – 2.25) | 1.60 (1.49 – 1.72) | 1.30 (1.21 – 1.39) | 1.45 (1.32 – 1.58) |
| Any | 1.26 (0.99 – 1.60) | 0.84 (0.67 – 1.06) | 0.79 (0.61 – 1.01) | 1.57 (1.10 – 2.23) | 1.28 (0.91 – 1.79) | 1.27 (0.86 – 1.88) | 1.26 (0.97 – 1.63) | 0.91 (0.72 – 1.14) | 0.82 (0.63 – 1.07) |
| P for interaction | <0.001 | 0.005 | 0.27 | 0.005 | 0.095 | 0.86 | <0.001 | 0.009 | 0.23 |
| Intensity of metformin use | |  |  |  |  |  |  |  |  |
| Median or below | 0.97 (0.65 – 1.44) | 0.99 (0.73 – 1.34) | 0.74 (0.52 – 1.05) | 1.22 (0.67 – 2.21) | 1.32 (0.83 – 2.09) | 1.26 (0.73 – 2.17) | 0.85 (0.55 – 1.33) | 1.01 (0.73 -1.38) | 0.77 (0.54 – 1.11) |
| Above median | 1.53 (1.13 – 2.07) | 0.70 (0.50 – 0.98) | 0.84 (0.59 – 1.20) | 1.87 (1.20 – 2.93) | 1.21 (0.74 – 1.97) | 1.26 (0.71 – 2.22) | 1.66 (1.20 – 2.29) | 0.80 (0.56 – 1.14) | 0.88 (0.60 – 1.28) |
| P for interaction | 0.010 | 0.079 | 0.95 | 0.072 | 0.42 | 0.71 | 0.002 | 0.21 | 0.95 |

Table 4. Effect of PSA-based screening on incidence on clinically significant prostate cancer as defined by Gleason score and TNM stage. Study population of 78,615 men from the Finnish Randomized Study of Screening for Prostate Cancer

|  | Gleason 7 | | | Gleason 8-10 | | | Advanced PCa* | | |
| --- | --- | --- | --- | --- | --- | --- | --- | --- | --- |
|  | nro of screening rounds participated | | | nro of screening rounds participated | | | nro of screening rounds participated | | |
|  | 1 | 2 | 3 | 1 | 2 | 3 | 1 | 2 | 3 |
|  | HR (95% CI) | HR (95% CI) | HR (95% CI) | HR (95% CI) | HR (95% CI) | HR (95% CI) | HR (95% CI) | HR (95% CI) | HR (95% CI) |
| Metformin | |  |  |  |  |  |  |  |  |
| n of users/non-users | 11,661/60,569 | 11,661/60,569 | 11,661/60,569 | 11,580/59,429 | 11,580/59,429 | 11,580/59,429 | 11,451/58,813 | 11,451/58,813 | 11,451/58,813 |
| None | 1.13 (0.97 – 1.31) | 0.80 (0.70 – 0.92) | 1.11 (0.96 – 1.27) | 1.06 (0.86 – 1.30) | 0.68 (0.56 – 0.83) | 0.81 (0.65 – 0.999) | 1.06 (0.83 – 1.35) | 0.48 (0.35 – 0.65) | 0.48 (0.33 – 0.70) |
| Any | 0.95 (0.60 – 1.52) | 0.63 (0.41 – 0.95) | 0.53 (0.33 – 0.83) | 1.28 (0.79 – 2.05) | 0.61 (0.38 – 0.996) | 0.66 (0.41 – 1.04) | 1.32 (0.70 – 2.48) | 0.37 (0.15 – 0.92) | 0.51 (0.22 – 1.20) |
| P for interaction | 0.11 | 0.20 | 0.054 | 0.96 | 0.70 | 0.74 | 0.89 | 0.55 | 0.44 |
| Intensity of metformin use | |  |  |  |  |  |  |  |  |
| Median or below | 0.40 (0.15 – 1.09) | 0.65 (0.36 – 1.16) | 0.45 (0.23 – 0.87) | 1.47 (0.77 – 2.81) | 1.04 (0.60 – 1.87) | 0.70 (0.36 – 1.37) | 2.29 (0.91 – 5.79) | 0.75 (0.22 – 2.55) | 0.39 (0.09 – 1.69) |
| Above median | 1.48 (0.86 – 2.54) | 0.61 (0.34 – 1.09) | 0.62 (0.33 – 1.16) | 1.11 (0.55 – 2.26) | 0.28 (0.11 – 0.70) | 0.78 (0.40 – 1.53) | 0.93 (0.39 – 2.23) | 0.21 (0.05 – 0.86) | 0.66 (0.23 – 1.89) |
| P for interaction | 0.011 | 0.90 | 0.68 | 0.65 | 0.026 | 0.96 | 0.31 | 0.21 | 0.93 |

Table 5. Effect of screening on prostate cancer –specific mortality among users and non-users of metformin. Study population of 78,615 men from the Finnish Randomized Study of Screening for Prostate Cancer

| Any antidiabetic medication use | Mortality by metformin usage HR (95% CI) | | |
| --- | --- | --- | --- |
| Number of participated screening rounds | 1 | 2 | 3 |
| none | 1.60 (1.28 – 2.01) | 0.51 (0.39 – 0.68) | 0.18 (0.11 – 0.32) |
| any | 2.11 (1.08 – 4.14) | 0.52 (0.21 – 1.33) | 0.46 (0.16 – 1.30) |
| P for interaction | 0.48 | 0.86 | 0.067 |
| Intensity of metformin use |  |  |  |
| Median or below | 2.36 (0.77 – 7.24) | 0.99 (0.28 – 3.46) | 0.92 (0.26 – 3.21) |
| Above median | 2.00 (0.86 – 4.64) | 0.30 (0.07 – 1.28) | 0.19 (0.03 – 1.43) |
| P for interaction | 0.92 | 0.24 | 0.16 |
