# Supplementary material for: Outcomes of prostate cancer screening among men using antidiabetic medication
Source: Sci Rep. 2021 Apr 1;11:7363. doi: 10.1038/s41598-021-86534-2 (PMC8016840; doi:10.1038/s41598-021-86534-2)
Supplement: Supplementary file 2 — Supplementary Information 2. [file 41598_2021_86534_MOESM2_ESM.docx]

Outcomes of prostate cancer screening among men using antidiabetic medication

Vettenranta A^1^, Murtola TJ^1,2^, Talala K^3^, Taari K^4^, Stenman U-H^4,5^, Tammela TLJ^1,2^, Auvinen A^6^

^1^ University of Tampere, Faculty of Medicine and Life Sciences, Tampere, Finland

^2^ Tampere University Hospital, Department of Urology, Tampere, Finland

^3^ Finnish Cancer Registry, Helsinki, Finland

^4^ Department of Urology, University of Helsinki and Helsinki University Hospital, Helsinki, Finland

^5^ Department of Clinical Chemistry, University of Helsinki, Helsinki, Finland

^6^ University of Tampere, Faculty of Social Sciences, Tampere, Finland

Corresponding author: Ms. Arla Vettenranta. Arvo Ylpön katu 34, PO box 100, 33014 University of Tampere, Finland. Phone: +358 3 355 111. E-mail: arla.vettenranta@tuni.fi

Median PSA levels by cancer type. Study population of 78,615 men from the Finnish Randomized Study of Prostate Cancer Screening.

| PSA median (IQ) | PCa overall | Gleason 6 or less | Gleason 7 | Gleason 8-10 | Advanced cancer |
| --- | --- | --- | --- | --- | --- |
| 1^st^ screening round | 2.74 (1.56-4.82) | 3.31 (2.05-5.56) | 2.13 (1.33-3.66) | 1.85 (1.05-3.49) | 2.13 (1.17-6.99) |
| 2^nd^ screening round | 1.45 (0.00-3.84) | 1.75 (0.00-4.39) | 1.67 (3.40) | 1.02 (0.00-2.75) | 0.00 (0.00-1.57) |
| 3^rd^ screening round | 0.00 (0.00-1.87) | na | 0.00 (0.00-3.21) | 0.00 (0.00-1-64) | na |

| PSA median (IQ) | PCa overall | Gleason 6 or less | Gleason 7 | Gleason 8-10 | Advanced cancer |
| --- | --- | --- | --- | --- | --- |
| 1^st^ screening round | 2.80 (1.60-4.98) | 3.38 (2.08-5.66) | 2.14 (1.35-3.76) | 1.87 (1.02-3.57) | 2.18 (1.01-7.70) |
|  | 2.22 (1.32-3.78) | 2.83 (1.73-4.71) | 2.01 (1.26-3.07) | 1.73 (1.10-3.15) | 2.07 (1.44-3.95) |
| 2^nd^ screening round | 1.48 (0.00-3.92) | 1.70 (0.00-4.40) | 1.73 (0.00-3.62) | 1.02 (0.00-2.73) | 0.00 (0.00-1.53) |
|  | 1.35 (0.00-3.35) | 1.76 (0.00-4.33) | 1.33 (0.00-3.07) | 1.00 (0.00-2.91) | 0.00 (0.00-2.16) |
| 3^rd^ screening round | 0.00 (0.00-1.77) | na | 0.00 (0.00-3.34) | 0.00 (0.00-1.41) | na |
|  | 0.00 (0.00-2.32) | 0.00 (0.00-2.27) | 0.00 (0.00-2.63) | 0.00 (0.00-2.08) | na |

Median PSA levels by cancer type and by antidiabetic medication use. Study population of 78,615 men from the Finnish Randomized Study of Prostate Cancer Screening.

* No antidiabetic medication use
