# Supplementary material for: Outcomes of prostate cancer screening among men using antidiabetic medication
Source: Sci Rep. 2021 Apr 1;11:7363. doi: 10.1038/s41598-021-86534-2 (PMC8016840; doi:10.1038/s41598-021-86534-2)
Supplement: Supplementary file 3 — Supplementary Information 3. [file 41598_2021_86534_MOESM3_ESM.docx]

Outcomes of prostate cancer screening among men using antidiabetic medication

Vettenranta A^1^, Murtola TJ^1,2^, Talala K^3^, Taari K^4^, Stenman U-H^4,5^, Tammela TLJ^1,2^, Auvinen A^6^

^1^ University of Tampere, Faculty of Medicine and Life Sciences, Tampere, Finland

^2^ Tampere University Hospital, Department of Urology, Tampere, Finland

^3^ Finnish Cancer Registry, Helsinki, Finland

^4^ Department of Urology, University of Helsinki and Helsinki University Hospital, Helsinki, Finland

^5^ Department of Clinical Chemistry, University of Helsinki, Helsinki, Finland

^6^ University of Tampere, Faculty of Social Sciences, Tampere, Finland

Corresponding author: Ms. Arla Vettenranta. Arvo Ylpön katu 34, PO box 100, 33014 University of Tampere, Finland. Phone: +358 3 355 111. E-mail: arla.vettenranta@tuni.fi

Figure 1.

Flowchart of the study population and analysis.

Study population

80,458 men

Baseline exclusion

remaining 80,139 men

Randomization

Control arm 48,273

Screening arm 31,866

Information about medication used: 78,165 men

Analyses

Antidm med. users Antidm med. non-users

SA

CA

CA

SA

HR

HR
